# A Dual Role of Heme Oxygenase-1 in Tuberculosis

**DOI:** 10.3389/fimmu.2022.842858

**Published:** 2022-02-25

**Authors:** Sen Yang, Jing Ouyang, Yanqiu Lu, Vijay Harypursat, Yaokai Chen

**Affiliations:** Division of Infectious Diseases, Chongqing Public Health Medical Center, Chongqing, China

**Keywords:** heme oxygenase-1, oxidative stress, iron, *Mycobacterium tuberculosis*, chemotherapy

## Abstract

Iron metabolism is vital for the survival of both humans and microorganisms. Heme oxygenase-1 (HO-1) is an essential stress-response enzyme highly expressed in the lungs, and catabolizes heme into ferrous iron, carbon monoxide (CO), and biliverdin (BV)/bilirubin (BR), especially in pathological conditions which cause oxidative stress and inflammation. Ferrous iron (Fe^2+^) is an important raw material for the synthesis of hemoglobin in red blood cells, and patients with iron deficiency are often associated with decreased cellular immunity. CO and BR can inhibit oxidative stress and inflammation. Thus, HO-1 is regarded as a cytoprotective molecule during the infection process. However, recent study has unveiled new information regarding HO-1. Being a highly infectious pathogenic bacterium, *Mycobacterium tuberculosis* (MTB) infection causes acute oxidative stress, and increases the expression of HO-1, which may in turn facilitate MTB survival and growth due to increased iron availability. Moreover, in severe cases of MTB infection, excessive reactive oxygen species (ROS) and free iron (Fe^2+^) due to high levels of HO-1 can lead to lipid peroxidation and ferroptosis, which may promote further MTB dissemination from cells undergoing ferroptosis. Therefore, it is important to understand and illustrate the dual role of HO-1 in tuberculosis. Herein, we critically review the interplay among HO-1, tuberculosis, and the host, thus paving the way for development of potential strategies for modulating HO-1 and iron metabolism.

## Introduction

Tuberculosis (TB), a highly infectious disease caused by *Mycobacterium tuberculosis* (MTB), is spread from person to person predominantly through air by droplet contact (1). After inhalation, the bacteria are phagocytosed by alveolar macrophages (2). During their contact with the invading pathogens, macrophages are activated to produce cytokines and high levels of reactive oxygen species (ROS) (3). These inflammatory responses are a major defense mechanism against invading pathogens; however, an excessive inflammatory response can result in accumulation of ROS and extensive tissue and organ damage (4). An imbalance in oxidant/antioxidant levels may lead to oxidative stress, which is likely to be a major mechanism causing cell and tissue injury, and even massive blood loss in TB patients (3). As a result, iron deficiency anemia is commonly observed among TB patients, which, in turn, is associated with decreased cellular immunity. It has been reported that tissue damage is associated with high levels of oxidation in tuberculosis patients (3, 5). ROS are highly reactive major cellular oxidants generated as byproducts of oxygen metabolism, and the accumulation of the ROS within cells may initiate cell necrosis (6, 7). Ferroptosis is a recently identified form of programmed cell death, differs from other cell death mechanisms, and results in an iron-dependent intracellular accumulation of ROS and lipid peroxides (8, 9). Amaral et al., reported in a recent study that excessive iron accumulation in MTB-infected cells can induce necrotic cell death *via* ferroptosis, which promotes tissue damage and facilitates bacterial dissemination (10).

**Figure 1:**
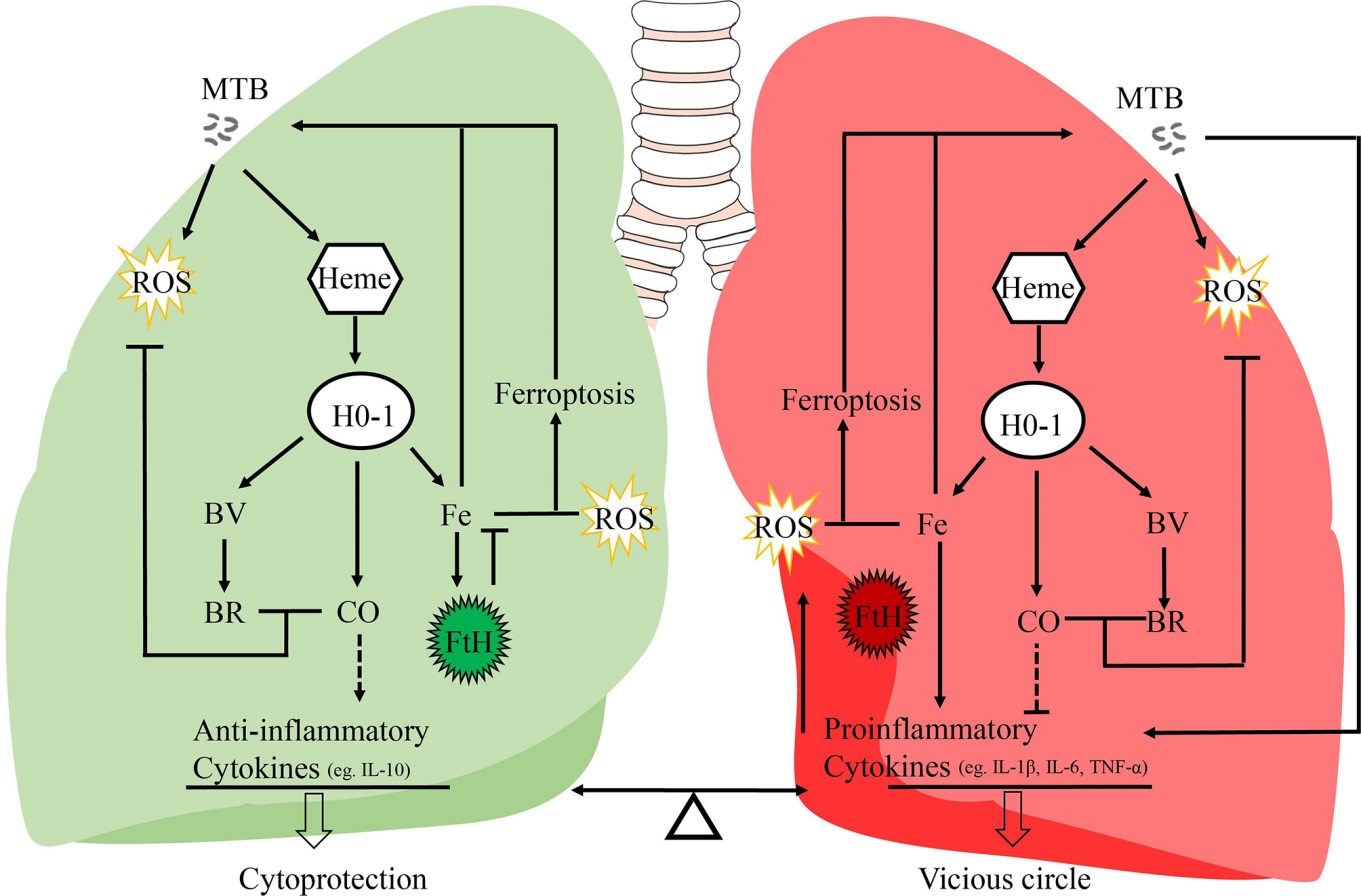
Potential mechanisms of the dual role of HO-1 in TB. It is generally assumed that this HO-1 and its products may act as a crucial regulator in inflammatory processes, and regulates the balance between proinflammatory and anti-inflammatory mediators. IL-10 is responsible for its anti-inflammatory properties; IL-1β, IL-6, and TNF-α are pro-inflammatory cytokines secreted by macrophages to initiate and regulate the progression of inflammation. MTB, Mycobacterium tuberculosis; HO-1, heme oxygenase-1; ROS, reactive oxygen species; BV, biliverdin; BR, bilirubin; FtH, ferritin heavy chain; CO, carbon monoxide; Fe, free iron.

Heme oxygenase-1 (HO-1) is a major antioxidant which is highly expressed in the lungs, and is activated by a variety of stress signals such as ROS and inflammatory mediators (11, 12). As a critical cytoprotective enzyme, HO-1 degrades free heme into free iron, carbon monoxide (CO), and biliverdin (BV), which, in turn, may be further converted to bilirubin (BR) (13). CO is known to possess anti-inflammatory, anti-oxidative, and anti-apoptotic effects (14). Otterbein et al., have demonstrated that CO exerts anti-inflammatory effects, in part, by increasing macrophage IL-10 production (15). Moreover, overexpression of exogenous HO-1 in the macrophages ensures a high level of endogenous IL-10 production by these cells (16) (**Figure 1**). BV, together with bilirubin, plays a protective role through their anti-inflammatory and antioxidant activities (17, 18). The precise role of HO-1 in the host response to MTB infection and disease progression is controversial. On the one hand, some studies suggest that HO-1 induction improves disease status in TB patients (3, 19, 20). The importance of HO-1 has been demonstrated in HO-1 knockout mice and human genetic HO-1 deficiency cases (21–25). Pro-inflammatory cytokines (IL-1β, IL-6, and TNF-α) secreted by macrophages in knockout mice are significantly increased, and these mice go on to develop a progressive inflammatory state (23). Previous studies have shown that proinflammatory cytokines such as IL-1β, IL-6, and TNF-α are key intermediaries of an over-responsive host-response reaction (26–28). IL-10 is a pleiotropic cytokine recognized for its inhibitory activity on a variety of immune functions (29). IL-10 exerts anti-inflammatory effects on macrophages and dendritic cells by suppressing production of inflammatory cytokines such as IL-1β, IL-6, and TNF-α (30) (**Figure 1**). Moreover, other studies have observed cytoprotective properties of HO-1, suggesting that modulation of HO-1 expression and activity could have potential therapeutic value (24, 25). On the other hand, several recent studies suggest that HO-1 might increase ferrous iron levels, which is associated with ferroptosis, and facilitate MTB survival and growth under specified disease conditions (31–33). In this review, we present information regarding recent advances with respect to HO-1, and critically discuss the role of HO-1 in human tuberculosis infection, paving the way for development of potential strategies to modulate HO-1 and iron metabolism.

## Molecular Characterization and Expression of HO-1

The HO protein family is an endoplasmic reticulum-associated enzyme group that is involved in the selective catabolism of free circulating heme. Three isoforms of HO are found in mammalian cells: an inductive form, HO-1, and two constitutive forms, HO-2 and HO-3 (13, 34, 35). The two main isoforms, HO-1 and HO-2, are products of two different genes, HMOX1 and HMOX2, which are located on chromosome 22 and chromosome 16, respectively (36). HO-3 is considered to be a pseudogene derived from HO-2 transcripts, and its function remains unclear (34). HO-1 and HO-2 differ in structure and tissue distribution (36). The enzyme HO-2 is a 36 kilodalton (kDa) protein that is highly detected in the brain, testis, and endothelial and smooth muscle cells in cerebral blood vessels (37). HO-1, also known as heat shock protein 32 (HSP32), is a 32kDa protein expressed in all mammalian tissues at basal levels, and is highly expressed in the lung, spleen, liver, bone marrow, and in senescent red blood cells (38).

**Figure 2:**
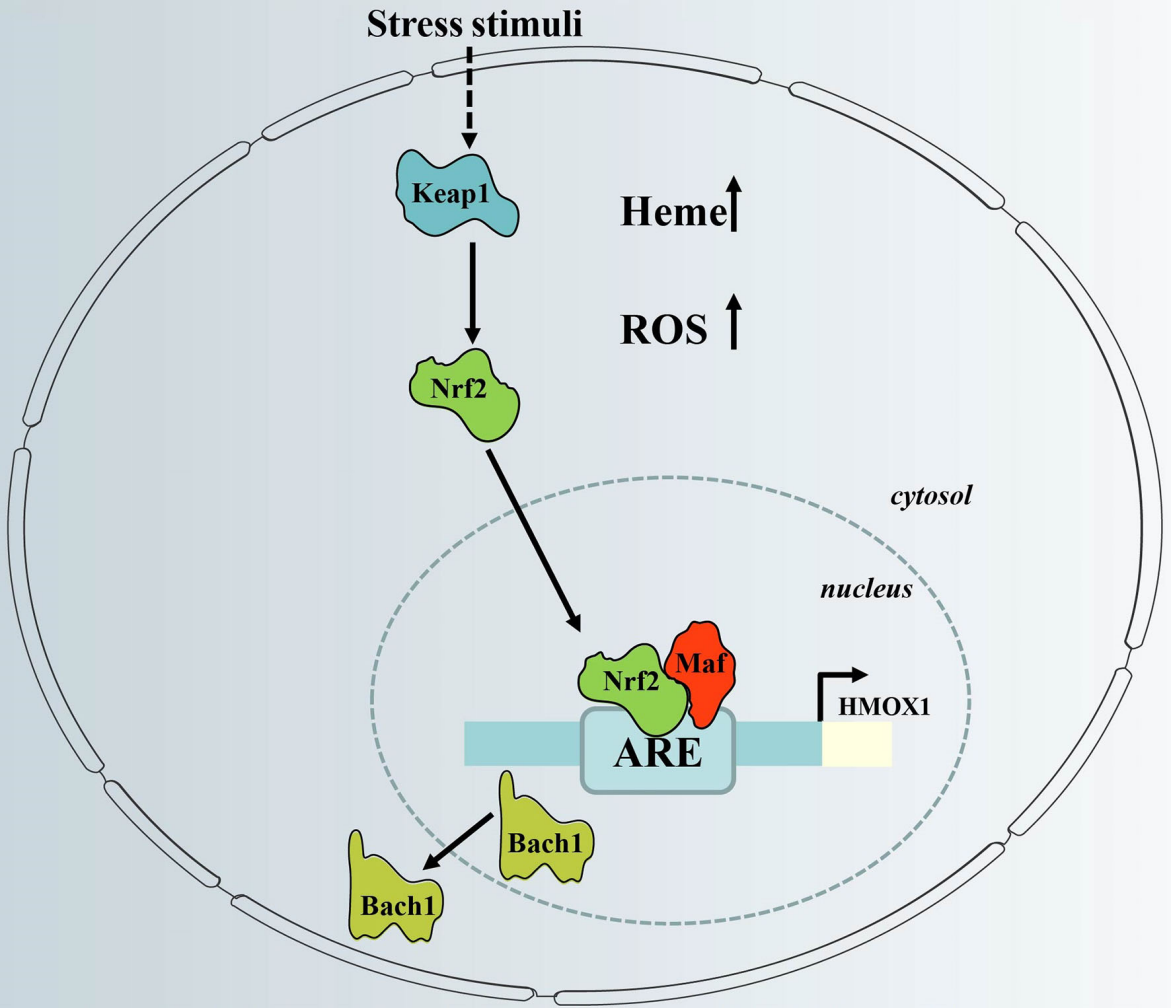
Regulation of HO-1 gene expression *via* the redox-dependent Keap1-Nrf2 system. HO-1 gene expression is regulated *via* inactivation of Bach1 and activation of Nrf2, which have counter-regulatory functions. When cellular heme levels are high, and in response to stress stimuli, Bach1 is removed from the HO-1 promoter. In addition, stress stimuli cause dissociation of Nrf2 from Keap1, which activates HO-1 gene expression after nuclear translocation *via* binding to HO-1 ARE. ARE, antioxidant response element; Keap1, Kelch-like ECH-associated protein 1; HMOX1, HO-1 gene; Bach1, BTB and CNC homologue 1; Nrf2, nuclear factor erythroid-related factor 2; ROS; reactive oxygen species.

The promoter region of the HMOX1 gene contains an antioxidant response element (ARE), which is important for the expression of HO-1, and consists of the BTB and CNC Homology 1 (Bach1) transcription factor, together with one of 3 small musculoaponeurotic fibrosarcoma oncogene homolog (Maf) proteins (MafF, MafG or MafK). Under normal conditions, BACH1 forms heterodimers with Maf, which binds Maf recognition elements (MAREs) within the promoter region of target genes to repress transcription (39). Under conditions of excess intracellular heme, heme binds to the heme-binding region of BACH1, which induces the dissociation of Bach1 from a heterodimeric repressor complex. The BACH1 molecule is then translocated into the cytoplasm, where it undergoes ubiquitination, followed by proteasomal degradation (40). This event allows Maf to form a dimer with nuclear factor erythroid-related factor 2 (Nrf2) *via* the same ARE region, resulting in transcriptional activation (41) (**Figure 2**). This process can be initiated by oxidative stress, which occurs, for example, when excessive ROS is produced (42).

Activation of Nrf2 requires dissociation from its partner, Kelch-like ECH-associated protein 1 (Keap1) (**Figure 2**). The Nrf2-Keap1 complex acts as a cellular sensor of xenobiotics, drugs, and radiation-induced ROS/electrophilic stress (43). Under oxidative stress, Nrf2 is released from its cytosolic inhibitor, Keap1, and translocates to the nucleus (44). It is also worth noting that the Keap1/Nrf2 module may not only be regulated by pro-oxidant stimuli. It is not clear whether Nrf2-dependent induction of HO-1 is part of a general Nrf2-regulated antioxidant protective response that includes other Keap1/Nrf2-regulated genes such as NAD(P)H: oxidoreductase or thioredoxin reductase-1 (45). Thus, the redox-dependent Keap1/Nrf2 system plays a central role in HO-1 induction in response to oxidative stress.

## Regulation of Intracellular Iron Distribution by HO-1

In general, heme iron is more bioavailable than free iron in mammals, and is the form in which iron is stored in the body (46). However, under various pathophysiological conditions, including sickle cell disease and malaria, excessive release of heme from liberated hemoglobin has pro-oxidant and cytotoxic effects (47). Malaria is a hemolytic disease characterized by high levels of free heme, and in responding to this stress, the expression of HO-1 is induced, which catabolizes excess heme into biliverdin, CO, and Fe^2+^ through degradation of the protoporphyrin IX ring of heme (48). Moreover, HO-1 also upregulates the iron-binding protein, ferritin, and the iron transporter, ferroportin (FPN), which exports labile iron to the extracellular milieu, resulting in reduced intracellular iron and oxidative stress (49, 50). Body iron is stored in the liver, in the form of ferritin and hemosiderin. When cellular iron exceeds requirements, the excessive iron is stored in a bioavailable form as ferritin, which protects cells from potentially toxic reactions catalyzed by iron (51). Ferritin thus has a dual function in facilitating both iron detoxification and acting as a reserve. Ferritin is a hetero-polymeric protein consisting of 24 subunits of two types, i.e., heavy (FtH, 21 kDa) chains and light (FtL, 19 kDa) chains (52). Ferritin-stored iron is believed to be utilized when cells become iron deficient; however, the precise mechanisms underlying the extraction of iron from ferritin have yet to be comprehensively elucidated (53). HO-1 has been reported to promote neutralization and detoxication of intracellular iron overload through inducing the FtH enzyme (54).

Given the essential functions of iron, and the toxicity associated with iron excess, humans have evolved specialized proteins and tightly regulated homeostatic mechanisms for the uptake, transport, storage, and export of iron, to provide adequate iron for essential biological processes, but also to limit the toxicity of iron excess (55). The major avenues for regulating systemic iron balance are in controlling dietary iron uptake, and iron release from hepatocytes and recycling macrophages. Dietary iron taken up by enterocytes can be stored largely in the form of ferritin (56). This allows iron to be stored in an inert form, and allows for a more controlled delivery of systemic iron. The majority of iron is delivered to the bone marrow for red blood cell (RBC) production, with smaller amounts being distributed to other tissues for fundamental cellular processes, and any excess iron is transported to the liver for storage (57). Iron recycling macrophages are a major storage site for iron in addition to liver hepatocytes, and provide a major source of daily iron. Systemic iron homeostasis is maintained predominantly by recycling iron from RBCs *via* reticuloendothelial macrophages. This specialized macrophage population phagocytoses old and damaged RBCs. RBCs are lysed, and iron is released from hemoglobin by HO-1. Iron can then be stored in ferritin and exported to the bloodstream by FPN, the only known mammalian iron exporter, which has been suggested to be a major gatekeeper by controlling iron entry into the bloodstream (58). The hormone, hepcidin, is a negative regulator of iron metabolism, and binds to FPN, inducing its internalization and degradation, and is involved in pathological regulation of iron in response to infection, inflammation, hypoxia, and anemia (59). Hepcidin controls the absorption of dietary iron as well as the distribution of iron between intracellular stores and extracellular fluids, including plasma (60). HO-1 contribution to iron homeostasis has been postulated. It has been reported that the patient with HO-1 deficiency exhibits both signs of inflammation and dysregulation of body iron homeostasis, including anemia and liver and kidney hemosiderosis, both induce hepcidin (61–63). Surprisingly, Kartikasari et al., observed low hepcidin levels in HO-1-deficient patients, suggesting increased need for iron in the bone marrow due to anemia (63). The decreased iron recycling, because of HO-1 deficiency, and subsequently, lower serum iron-transferrin levels may, in part, have contributed to the observed low hepcidin levels (63).. HO-activity was not observed to have a direct modulating effect on expression of HAMP, the gene that encodes for hepcidin (63). Charlebois et al., confirmed that hepatocellular HO-1 has no physiological effect on hepcidin regulation *via* the inflammatory pathway (64). The preceding findings indicate that dysregulation of iron homeostasis in HO-1 deficiency is the result of both defective iron recycling and erythroid activity-associated inhibition of hepcidin expression.

## Pro-Oxidant and Pro-Inflammatory Effects of MTB

Inflammation is a primary immune system reaction, and as the first response of host defense, free radicals are produced by inflammatory cells to eliminate pathogens (65, 66). Tuberculosis, like other chronic inflammatory diseases, is characterized by production of free radicals such as ROS (67). ROS play an important role in host defenses against MTB, and are produced by macrophages (68). However, ROS accumulation is considered potentially harmful due to their contribution to oxidative stress and tissue damage, resulting in damage to host immunity, especially in people with impaired antioxidant capacity, such as patients with HIV infection (69). Free radical generation in excess of the antioxidant capacity of the host may lead to oxidative stress, which can be amplified by tissue damage and cell death (67, 70). Cell damage and death leads to increased free radical production and decreased antioxidant capacity, and eventually a vicious cycle is established (71).

The clinical manifestations of tuberculosis in humans and animals are proportional to the severity of pulmonary and extra-pulmonary inflammation (67, 72). The tuberculous lesion is primarily composed of macrophages and granulocytes during the early stages of MTB infection (67). Jack et al., showed that free oxygen radicals are released from polymorphonuclear leukocytes and macrophages during inflammation (73). A recent study by Scharn et al., demonstrated that the development of tuberculous pulmonary lesion necrosis correlates with an increase in macrophages and granulocyte influx into the lung (33). In addition to co-localization of inflammatory cells to sites of necrosis, these cells eventually release their cytoplasmic contents, thus resulting in extracellular accumulation of iron and copper, both of which catalyze the production of free radicals (74, 75).

It is also known that inflammatory mediators and immunity-related factors can directly or indirectly produce red cell hemolysis, leading to increased extracellular hemoglobin (68, 76). Excess free heme, which is released from heme proteins under oxidative stress, is highly cytotoxic *via* the production of free radicals (77–79). Furthermore, free heme sensitizes non-hematopoietic cells to undergo programmed cell death, particularly in the course of infection by intracellular pathogens such as *Mycobacterium tuberculosis* (54, 68, 80). This deleterious effect of free heme is most probably driven by Fe (81, 82); however, it is not clear whether Fe must first be released from heme or must still be contained within the protoporphyrin ring of heme for cytotoxicity to occur, or whether both of the preceding scenarios are able to induce cytotoxicity (81, 82).

It is well known that hemoptysis and pulmonary hemorrhage, which frees heme, are typical clinical features of acute human TB (80). Hence, acute elevation in plasma free heme levels may overwhelm the antioxidant function of HO-1, and contribute to the immunopathology of TB by dysregulation of oxidative, inflammatory, and iron homeostasis (3, 80). In a cross-sectional study by Madebo et al., the markers of free radical damage in the peripheral circulation of patients with active tuberculosis were found to be elevated (83). These results were further confirmed by Taha et al., as active pulmonary tuberculosis was associated with oxidative stress (84). Moreover, oxidative stress is associated with the pathogenesis of lung fibrosis and dysfunction in tuberculosis patients even following effective antimicrobial therapy (73, 83).

## Iron Acquisition of Mycobacterium tuberculosis

MTB is transmitted to human hosts *via* the respiratory route and persists primarily within lung alveolar macrophages as a facultative intracellular pathogen (85, 86). Within the macrophage, MTB creates the environment required to establish infection by disrupting phagolysosome maturation, which is an iron-dependent process (82, 86). Iron is an essential nutrient for humans and almost all pathogenic microorganisms (82). MTB may utilize multiple pathways to obtain host iron (87–91). It is an important survival strategy for MTB to produce a diffusion-and-capture system to capture iron, where MTB expresses siderophores known as mycobactins, which diffuse out of phagosomes, chelates iron, and then re-enters the phagosome (88). Luo et al., revealed that MTB also can acquire iron *via* endocytosis of transferrin (Tf), the main iron carrier in plasma, which means that the intracellular pool of host iron can be accessed by MTB (87). Moreover, MTB can manipulate host cell iron homeostasis to support its growth by attenuating expression of the iron export protein FPN to increase intracellular iron content (87).Recent evidence has revealed that MTB employs the heme/hemoglobin uptake system of the host macrophage to obtain host iron in early endosomal phagosomes (89–91).

Considering the absolute requirement for iron by almost all human pathogens, nutritional immunity aims to limit iron availability to invading microorganisms, and plays an important role in the innate immune system (81, 92). To limit MTB iron acquisition, pro-inflammatory cytokines, such as IFN-gamma, downregulate transferrin receptors of macrophages, which decrease iron availability in late endosomes (93). Additionally, macrophages synthesize hepcidin in response to infectious agents, allowing for modulation of iron availability at the infectious focus (59, 94, 95). For intracellular agents, such as MTB, the hepcidin-ferroportin axis may play an important role to promote outflow of iron from the infected cell and deprive MTB of the iron required for it to divide and multiply (96).

Major hepcidin regulators include iron, erythropoietic drive, and inflammation (55). In particular, iron loading stimulates hepcidin expression, which can sequester iron from pathogenic microorganisms. Conversely, iron deficiency states such as anemias, inhibit hepcidin as a feedback mechanism to maintain normal body iron levels; however, this also leads to hypoferremia and iron restricted erythropoiesis in chronic inflammatory diseases (42). These results were observed by Chinta et al., where pulmonary hemorrhage and excessive iron deposition were observed in pulmonary TB patients (3).

## The Dual Role of HO-1 in TB

HO-1, as a major antioxidant which is highly expressed in lung tissue, plays a controversial role in human tuberculosis (3, 97). Previous studies have shown that the induction of HO-1 exerts protective effects in cells (48, 97, 98). Conversely, Costa et al., reported that pharmacological inhibition of HO-1 contributes to a substantial reduction of MTB burden, implying that HO-1 plays a pathogenic role rather than a protective in TB (32).

HO-1 is commonly regarded as a cytoprotective molecule during the infection process. HO-1 levels have been shown to distinguish active TB from latent TB and successfully-treated TB patients, and the increased HO-1 levels observed in plasma derives from injured tissues, and is strongly associated with bacterial burden (99, 100). HO-1 levels of latent MTB carriers were found to be comparable to healthy people, while high levels of serum HO-1 were found in patients with active tuberculosis, and HO-1 levels returned to baseline following successful treatment (97, 99). Andrade et al., showed that HO-1 level is a biomarker of active disease, and can be used to monitor the clinical condition of TB patients after receiving chemotherapy (99). Additionally, HO-1 levels positively correlate with plasma IL-10 levels and negatively correlate with TNF-alpha levels, suggesting that increased plasma levels of HO-1 may be involved in the regulation of inflammatory responses in active pulmonary tuberculosis (97, 101).

HO-1 plays a critical protective role in oxidant-induced acute lung injury (ALI), which is known to be a potent pro-oxidant and pro-inflammatory state (102). Studies have shown that oxidative stress biomarkers such as total oxidative status, malondialdehyde, and lipid peroxidation increase in pulmonary tuberculosis patients (3, 67). HO-1 catalyzes the oxidation of heme to generate iron, CO, and biliverdin, which have important anti-oxidant, anti-inflammatory, and anti-apoptotic properties (13). In addition, an increase in the intracellular free heme concentration due to massive hemorrhage is observed during active TB, and the cytoprotective role of HO-1 induction can be explained by the removal of pro-oxidant free heme in human TB patients (3, 97). Recent studies have revealed that decreased HO-1 levels positively correlate with significantly elevated ROS/RNS in neutrophils and macrophages isolated from severely damaged regions compared to healthy regions, strongly suggesting a cytoprotective role for HO-1 (3). Furthermore, HO-1 deficient mice are more susceptible to MTB infection, have increased bacterial loads, and higher mortality rates, whereas up-regulation of HO-1 has been shown to offer protection against infection, indicating the critical importance of HO-1 in controlling infection by MTB (20–23).

However, several groups have reported that treatment with SnPPIX (an HO-1 enzyme inhibitor) enhanced control of bacterial replication *in vitro* and decreased production of the anti-inflammatory cytokine, IL-10 (31–33). Additionally, they believe that HO-1 expression increases iron availability in activated macrophages, which is beneficial for growth of MTB (32, 103). Heme can be utilized as an abundant source of iron for replication by intracellular pathogens, directly enhancing their survival and growth inside host cells, including phagocytes (90). Although HO-1 inhibition has resulted in decreased bacterial burdens, no increase in survival has been observed (32). With respect to the preceding study, the period allocated for follow-up post-infection bacterial counts was relatively short, and it may require a longer period of follow-up to accurately determine the full effect of treatment, since TB is a chronic disease. Another limitation of the Costa et al., study is a lack of corresponding pathological data that could be utilized to relate the MTB bacterial load results to specific alterations in patient pathological states.

**Figure 3:**
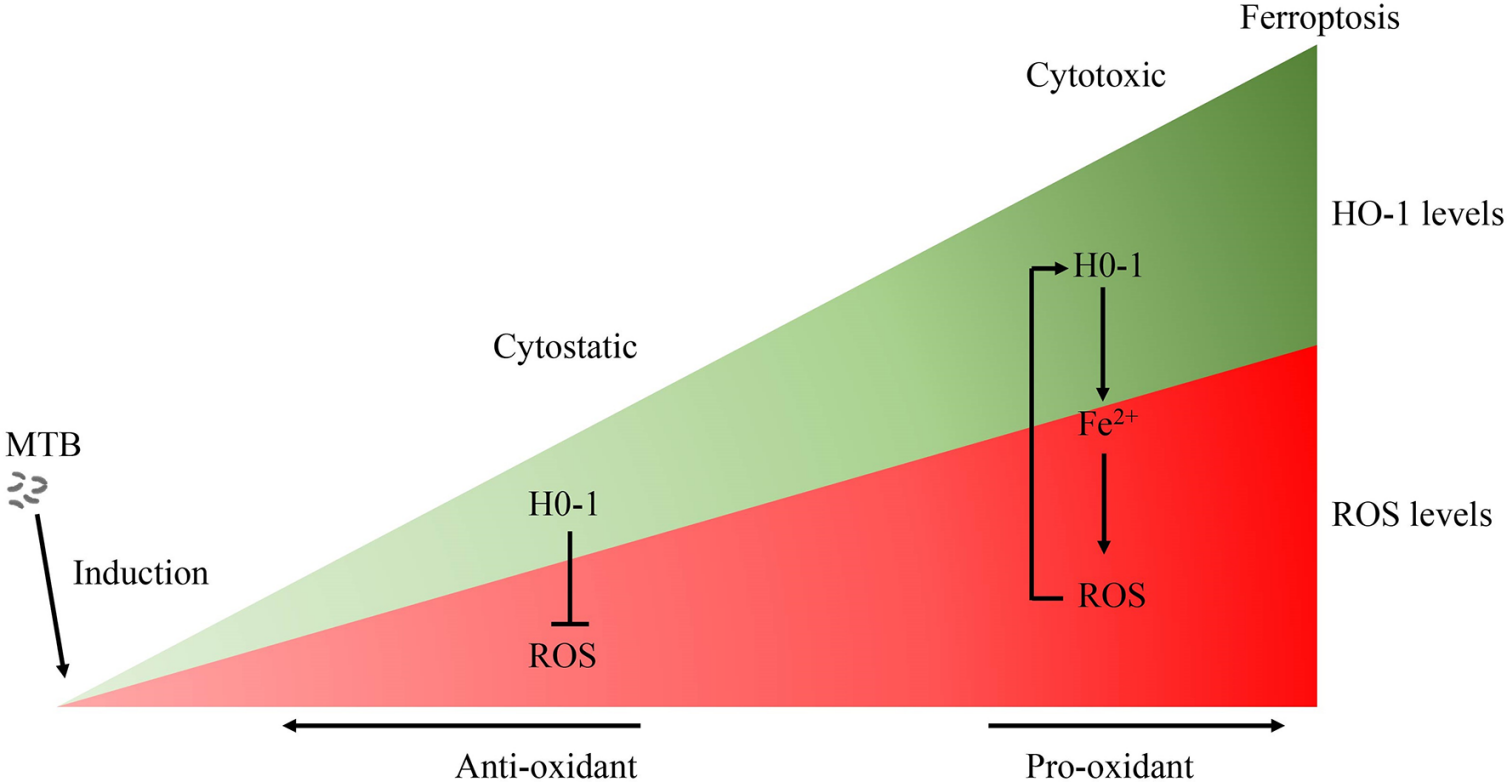
The role of HO-1 in MTB infection. Free heme is highly cytotoxic *via* the production of free radicals. Accordingly, when HO-1 is activated moderately, HO-1 exerts a cytoprotective effect by neutralizing ROS. Conversely, the large amounts of excessive free heme released during the late stages of tuberculosis overwhelms the cytoprotective effects of HO-1, thereby contributing to oxidative stress and excessive ROS production. The over-activation of HO-1 becomes detrimental due to the excessive labile Fe^2+^, along with ROS overload, leading to oxidative-cell death (ferroptosis), which contributes to the spread of infection. MTB, Mycobacterium tuberculosis; HO-1, heme oxygenase-1; ROS, reactive oxygen species; Fe, free iron.

The effects of HO-1 are dependent on cellular iron homoeostasis (**Figure 1**). Ferrous iron, a product of HO-1-mediated heme degradation, is generally considered to be a pro-inflammatory agent, and an essential nutrient for MTB (104). The increased ferrous iron is diverted from the pro-oxidant Fenton reaction because it is sequestered by cellular ferritin, which is co-induced with HO-1 during the early stages of the disease (54). Recently, Reddy et al., observed that hemosiderin deposits are seen in granulomatous lesions *via* iron van Gieson staining in human tuberculous lungs, suggesting that there may be little free iron available for MTB growth in tuberculous lungs (80). Thus, when the human host is able to establish an iron-deprived environment for MTB at the early stage of infection, HO-1 induction occurs as a protective factor against oxidant-mediated cellular injury (**Figures 1**, **3**).

Although there is no direct evidence to support clear causality, the distribution of iron within human lung tissue shows that iron homeostasis of distinct microanatomic locations is severely disrupted by MTB infection, and this may contribute to lung immunopathology, suggesting that the ability of the host cell to maintain intracellular iron homeostasis is disrupted (3, 75). The large amounts of hemoglobin and heme released during the late stages of tuberculosis, and the presence of excessive free heme, overwhelms the cytoprotective effects of HO-1, thereby contributing to oxidative stress and excessive ROS production (3, 80). Moreover, over-activation of HO-1 increases labile Fe**^2+^**, and the binding capacity of ferritin is disturbed by iron-catalyzed oxidative stress, causing an uncontrolled release of iron, finally resulting in enhancement of lipid peroxidation and cell death, which accelerates the spread of infection. Iron produced by the decomposition of heme by HO-1 further promotes pro-oxidizer-mediated inflammation, which leads to a vicious circle of HO-1 over activation resulting in labile Fe^2+^, which, in turn, results in excessive ROS, leading to oxidative cell death and a proinflammatory response. All components of this vicious circle thus exacerbates the progression of immunopathology and disease in chronic tuberculosis (75, 105) (**Figure 1**).

## The Modulators and Inducers of HO-1

It is a well-established fact that oxidative stress is a major inducer of HO-1 expression and is closely linked to inflammation (61). However, other stressful conditions also been reported to trigger HMOX1 transcription, such as pathogen-associated molecular patterns (PAMPs), danger-associated molecular patterns (DAMPs), and several different upstream signaling molecules and transcription factors (106, 107).

Previous reports have indicated that mitogen-activated protein kinases (MAPKs) are commonly activated in response to the stimuli referred to above (61, 106, 107), and play a critical role in HO-1 up-regulation (106–108). Three major subfamilies of MAPK, including p38 MAP kinase, the extracellular regulated kinases (ERK), and c-Jun N-terminal protein kinase (JNK), have been regarded as important pathways for the induction of HO-1 gene expression (108). PI3K signaling is known to regulate cellular proliferation and growth, and play a critical role in triggering inflammatory reactions through the activation of the downstream protein kinase, Akt (109). Zhuang et al., have reported that HO-1 expression may be up-regulated by rhein to protect IEC-6 cells against oxidative damage, *via* the PI3K/Akt pathway (110). In addition, Chen et al., found that PI3K/Akt activation not only up-regulates HO-1 gene expression, but also that the protective effects of this pathway might be linked to the beneficial effects of HO-1 (111). Previous reports have shown that other signaling pathways, such as the protein kinase A (PKA) pathway and the protein kinase C (PKC) pathway, have been associated with HO-1 up-regulation (112). It is important to point out that, in the regulation of HO-1, these signaling cascades are probably dependent on the specific cell types and specific inducers.

Several redox-sensitive transcription factors can be indirectly modulated by the various proteins mentioned above, such as MAPKs (61), PI3K (109), and other protein kinases (108), under a wide range of stressful conditions, leading to HO-1 regulation. As mentioned previously (41) the Nrf2 signaling pathway can regulate HO-1 gene transcription by targeting AREs. Nrf2/HO-1 activation induced by 4-Ketopinoresinol may be suppressed by inhibition of the PI3K-Akt pathway using chemical inhibitors or RNA interference, suggesting that the cytoprotective effect against oxidative damage may be eliminated (111). Other transcription factors, such as heat-shock factors (HSFs), activator protein-1 (AP-1), nuclear factor-kappa B (NF-kB), and hypoxia-induced factor 1 alpha (HIF1α) have also been reported to regulate HO-1 expression (108, 113).

Anti-tuberculosis drug-induced hepatotoxicity is one of the leading adverse drug reactions during the course of tuberculosis treatment, and poses a considerable challenge to clinicians and researchers (114). Previous studies suggest that anti-TB drugs may cause hepatotoxicity by generating toxic intermediaries *via* drug metabolism reactions (115). These reactive metabolites could induce the production of excessive reactive oxygen species (ROS), leading to lipid peroxidation and cell death (115). Otterbein et al., have demonstrated that co-administration of Isoniazid (INH) and rifampin (RIF) to HepG2 cells could induce expression of the cytoplasmic HO-1 protein during the early hours of drug treatment, with minimum loss of cell viability and cell death, and induction of HO-1 by hemin chloride could reverse the drug induced liver injury (116). This could be a potential target for a future therapeutic option to counteract INH-RIF drug-induced liver injury. Moreover, Lee et al., reported that rifampicin effectively protects the liver against cellular oxidative stress by activating AMPKα-induced activation of the Nrf2 transcription factor, with subsequent upregulation of HO-1 (117).

Several plant-based extracts and drugs have been reported to induce HO-1 expression in a variety of cells (118). The *Sagittaria sagittifolia* L-polysaccharide (SSP) is a purified form of a homogeneous polysaccharide isolated from the root tubers of *S. sagittifolia*. Wang et al., showed that, in mice, SSP significantly alleviates the hepatotoxicity induced by co-administration of INH and RIF, which is mainly attributable to an increase in expression of Nrf2 and its downstream enzyme HO-1, induced by SSP (119). Curcumin, a polyphenolic compound isolated from turmeric, has also been found to exert significant anti-inflammatory activity as a non-cytotoxic HO-1 inducer in vascular smooth muscle cells (VSMCs), endothelial cells, astrocytes, and macrophages (120, 121). Kundu et al., studied the effect of the coumarin derivative, fraxetin, on HO-1 expression in HaCaT cells, and found that fraxetin induced HO-1 expression through activation of the Akt/Nrf2 or the AMPK/Nrf2 pathways (122). Martin et al., has demonstrated the antioxidant effect of the herb-derived phenol, carnosol, which induced HO-1 expression at both mRNA and protein levels. Moreover, carnosol increased the nuclear levels of Nrf2, a transcription factor regulating AREs (123). Several HO-1-inducing compounds derived from natural sources have been also been reported to treat inflammatory conditions, including quercetin (124), resveratrol (125), anthocyanins (126), celastrol (127), and sulforaphane (128). Moreover, older repurposed drugs with novel uses have been reported. Dunigan-Russell et al., found that auranofin, an FDA-approved drug for rheumatoid arthritis, which was used to inhibit thioredoxin reductase-1 (TrxR1), was observed to activate Nrf2 responses to augment gene expression of HO-1, and thus prevent lung injury in acute respiratory distress syndrome (ARDS) (129). Phorbol myristate acetate (PMA), a strong inducer of NF-kB activity, has been reported by Naidu et al., to induce expression of HO-1 in murine primary astrocytes (130). Thus, using active components from medicinal plants and the use of repurposed old drugs have been shown to induce the expression of HO-1, and could possibly lead to new therapeutic strategies for oxidative stress-related diseases (61, 106). Over the past decade, the beneficial effects of HO-1 induction have been extensively studied and reported, as shown in **Table 1**.

**Table 1:** Natural source, evaluated model of diseases, reported effect, and pathway of natural compounds and drugs.

Compound	Source	Effect	Pathway	Model	References
SSP	S. sagittifolia	↑gene and protein expression of Nrf2 and HO-1	Nrf2/HO-1	mice	(119)
Curcumin	Curcuma longa	↑HO-1 mRNA in the liver, ↓oxidative stress and inflammation	Nrf2/HO-1 and TGF-β1/Smad3	CCl_4_-induced acute liver injury in mice	(131)
Fraxetin	Coumarin derivative	↑mRNA and protein expression of HO-1	Akt/Nrf2 or AMPKα/Nrf2	HaCaT human keratinocytes	(122)
Carnosol	Herb rosemary	↑HO-1 expression at both mRNA and protein levels	ERK, p38, JNK and PI3K	PC12 cells	(123)
Quercetin	Foods of plant origin	↑HO-1 expression at both transcription and translation levels	p38MAPK	RASMCs	(124)
Resveratrol	Fruits and vegetables	↑HO-1 expression, ↓Aβ_1-42_-induced oxidative stress	PI3K/AKT/Nrf2	Aβ_1-42_-induced cytotoxicity in PC12 cells	(125)
Anthocyanins	Vegetables, flowers, and fruits	↑Nrf2 and HO-1 mRNA levels, ↑GSH and GPx activity, ↓MDA and ROS levels	Nrf2/HO-1	Diabetes-induced oxidative stress and inflammation in rat retinas	(126)
Celastrol	Thunder God Vine and Celastrus regelii plant	↑Nrf2 and HO-1 expression, ↓mRNA levels of macrophage M1 biomarkers	Nrf2/HO-1, MAPK and NF-κB	Diet-induced obese C57BL/6N male mice	(127)
Sulforaphane	Broccoli	↑HO-1 expression, ↓inflammatory response	PI3K/Akt	MALP-2-induced pulmonary inflammation in mice	(128)
Auranofin	an FDA- approved drug	↑Hmox1 mRNA levels	Nrf2	Nrf2^KO1.3^ and Nrf2^KO2.2^ mtCCs	(129)
PMA	Natural compounds that activate PKC	↑HO-1 gene expression	NF-κB, p38 MAPK and CK2	RAW264.7 monocytes and p65(-/-) mice	(130)

Only the part of studies where HO-1 induction was evaluated are listed.

## Conclusion

Current anti-TB antibiotic therapy mainly targets actively replicating bacteria, and accelerates bacterial clearance; however, they have little effect on improving disease pathology. In addition, poor patient compliance caused by the prolonged treatment duration can lead to treatment failure, and induce the emergence of multi-drug and drug resistant *Mycobacterium tuberculosis* strains (132). Therefore, the identification and study of effective pharmacological targets to improve the pathology related to tuberculosis can be an important and beneficial adjunct to the limited pharmacological treatment strategies for TB that are currently available. As a component of the nutrition of the human body and the nutritional immunity of host, iron homeostasis of host cells can withhold iron from *Mycobacterium tuberculosis* (49, 54). HO-1 plays an important role in iron homeostasis and the antioxidant system as a cytoprotective enzyme to control oxidative stress-induced cellular damage. However, the dual role of HO-1 in tuberculosis may depend on different pathological conditions (**Figure 3**): On the one hand, HO-1 plays a mainly cytoprotective role in the early stages of the disease, and on the other hand, HO-1 can potentially be cytotoxic due to accumulation of large amounts of heme, and the human host may have an impaired ability to maintain iron homeostasis during the later stages of *Mycobacterium tuberculosis* infection. HO-1 is highly inducible in response to a variety of stimuli, such as oxidative stress, hypoxia, bacterial lipopolysaccharide (LPS), cytokines, and its substrate heme. Targeted induction of this powerful enzyme may be a useful therapeutic strategy to supplement conventional pharmaceutical anti-TB therapy, and to help serve a protective function by improving oxidative stress disorders, to decrease lesional inflammatory cell infiltration, to further support anti-inflammatory activity, and to induce tissue repair at later stages of MTB infection.

## Author Contributions

SY and JO wrote the initial draft of the manuscript. YL and VH critically revised the manuscript. YC conceived and designed the study, and provided critical revision of the manuscript. YC supervised the work. All authors contributed to the article and agreed to the published version of the manuscript.

## Funding

This work was supported by Chongqing Talent Cultivation Program (cstc2021ycjh-bgzxm0275), the Joint Medical Research Projects of Chongqing Municipal Health Committee and Chongqing Municipal Science and Technology Bureau (2020MSXM097, 2022QNXM032), the medical scientific research project of Chongqing Health Commission(2022WSJK006) and Scientific Research Project of Chongqing Public Health Medical Center (2022QNKYXM01).

## Conflict of Interest

The authors declare that the research was conducted in the absence of any commercial or financial relationships that could be construed as a potential conflict of interest.

## Publisher’s Note

All claims expressed in this article are solely those of the authors and do not necessarily represent those of their affiliated organizations, or those of the publisher, the editors and the reviewers. Any product that may be evaluated in this article, or claim that may be made by its manufacturer, is not guaranteed or endorsed by the publisher.
